# Interplay of NK cells and monocytes in vascular inflammation and myocardial infarction

**DOI:** 10.3389/fphys.2014.00295

**Published:** 2014-08-14

**Authors:** Maike Knorr, Thomas Münzel, Philip Wenzel

**Affiliations:** Department of Medicine 2 and Center for Thrombosis and HemostasisUniversity Medical Center Mainz, Mainz, Germany

**Keywords:** monocytes, nk cells, vascular inflammation, atherosclerosis, myocardial infarction

## Abstract

Inflammatory monocytes and macrophages have been identified as key players in the pathogenesis of atherosclerosis, arterial hypertension, and myocardial infarction (MI). They become powerful mediators of vascular inflammation through their capacity to secrete and induce the production of proinflammatory cytokines, chemokines and adhesion molecules and through the production of reactive oxygen species mainly via their NADPH oxidase. Importantly, a crosstalk exists between NK cells and monocytes that works via a feedforwad amplification loop of T-bet/Interferon-gamma/interleukin-12 signaling, that causes mutual activation of both NK cells and monocytes and that fosters recruitment of inflammatory cells to sites of inflammation. Recently, we have discovered that this crosstalk is crucial for the unrestricted development of angiotensin II (ATII) induced vascular injury in arterial hypertension, the most important risk factor for atherosclerosis and cardiovascular disease worldwide. In this review, we will also discuss possible implications of this interplay between NK cells and monocytes for the pathogenesis of coronary atherosclerosis and myocardial infarction and potential therapeutic options.

## Role of monocytes/macrophages in atherosclerosis, coronary artery disease, acute coronary syndrome and vascular inflammation

Myocardial infarction (MI) is one of the leading causes of morbidity and mortality in western societies (Hausenloy, 2009). Many risk factors like arterial hypertension, diabetes mellitus or lipid metabolism disorders can result alone or in combination in stenosis of the coronary blood vessels. Within the last years multiple genetic factors and the influence of the immune system were described in the pathogenesis of atherosclerosis. Innate immunity pathways have long been suspected to contribute to the initiation and progression of atherosclerosis (Shibata and Glass, 2009). Hence, atherosclerosis and its sequela can be considered as both a chronic inflammatory disease and as a metabolic disorder.

### The sequence of neutrophils and monocytes

Polymorphonuclear leukocytes (PMNs, reflecting mainly neutrophil granulocytes) dominate the initial influx of leukocytes to sites of acute infection and inflammation followed by emigration of monocytes. In 1968 Ward et al. described first that there exists a causal link between initial extravasation of PMNs followed by later emigration of monocytes (Ward, 1968). Gallin et al. identified that in patients who suffer from specific granule deficiency had a reduced effect on chemotactic effect on monocytes (Gallin et al., 1982). In different mouse studies it seemed that recruited PMNs trigger monocyte recruitment by releasing soluble factors (Doherty et al., 1988; Fillion et al., 2001; Janardhan et al., 2006). Different granule proteins of PMNs, for example cathepsin and human neutrophil peptide 1–3, with monocyte chemotactic activity could be identified (Territo et al., 1989; Chertov et al., 1997).

Peripheral blood monocytes are a heterogeneous group of circulating leukocytes, which can be distinguished in different subsets with diverse functions. These subsets have different phenotypes in mice and humans (Tacke et al., 2007; Ley et al., 2011). Geissmann et al. identified two subsets in murine blood that can be discriminated by the receptor for the chemokine fractalkine, the CX_3_CR_1_: The short lived CX^lo^_3_CR_1_CCR2^+^GR1^+^ (more specifically, Ly6C^hi^) cells, which are pro-inflammatory, are chemoattracted by MCP-1 and migrate to sites of inflammation and a CX_3_CR^hi^_1_CCR2^−^GR1^−^ subset (Ly6C^lo^) not attracted by inflamed tissues, that was later termed reparative. The CX_3_CR^lo^_1_CCR2^+^GR1^+^ cells correspond to CD14^hi^CD16^−^ monocytes, whereas the latter were equivalent to CD14^lo^CD16^+^ monocytes in humans (Geissmann et al., 2003). They express different levels of chemokine receptors and cytokines depending on their function (Weber et al., 2000). CD14^hi^CD16^−^ monocytes produce high levels of pro-inflammatory cytokines such as tumor necrosis factor alpha (TNF-α) and low levels of anti-inflammatory cytokines such as IL-10. In contrast, CD14^lo^CD16^+^ monocytes have been shown to produce high levels of IL-10 and low of TNF-α (Patino et al., 2000; Belge et al., 2002; Skrzeczynska-Moncznik et al., 2008). Shortly after acute MI in patients CD14^hi^CD16^+^ monocytes were increased and decreased at day 7 (Tsujioka et al., 2009). All subsets of monocytes can differentiate into macrophages after being recruited to lesions and then can be found in all tissues, where they have functional diversity (Wynn et al., 2013). The macrophages can be divided in M1 and M2 macrophages, M1 are activated by interferon gamma (IFN-γ) and have a more inflammatory phenotype by producing nitrogen intermediaries and pro-inflammatory cytokines (IL-1β and TNF-α) and participating in TH1 polarization. M2 macrophages act more in an anti-inflammatory way (IL-12^low^ and IL-10^high^) and participate in TH2 responses (Murray and Wynn, 2011). In mouse models of atherosclerosis, an infiltration of M2 macrophages in the early lesions could been identified, whereas M1 macrophages were predominant in advanced stages of the disease (Khallou-Laschet et al., 2010). Interestingly, it was recently shown, that experimental MI accelerates atherosclerosis in high fat diet fed ApoE^−/−^ mice driven by inflammatory Ly6C^hi^ monocytes (Dutta et al., 2012). This study provided a mechanistic explanation for the fact that history of MI is a risk factor for cardiovascular disease. It also highlightens the essential role of monocyte driven vascular inflammation for coronary atherosclerosis and MI.

### The inflammatory response in cardiac ischemic injury

**Figure 1:**
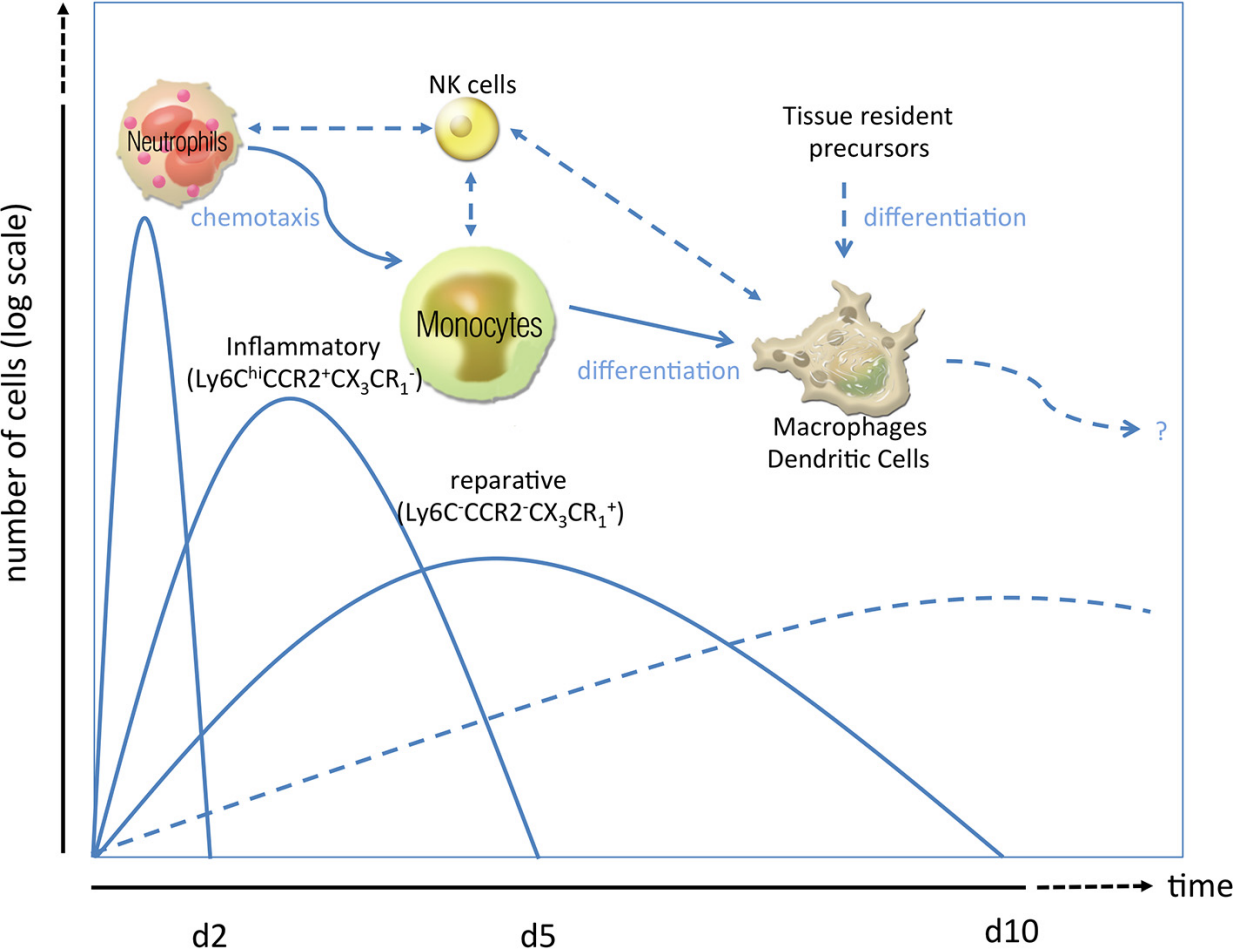
**Phases of the inflammatory response in ischemic injury**. After ischemic injury, e.g., myocardial infarction, a sequence of inflammatory events has been proposed. As first line of defense, neutrophil granulocytes rush in clearing debris and paving the way for monocytosis by specific chemotactic patterns (Soehnlein et al., 2008; Wantha et al., 2013). Neutrophils are followed by inflammatory Ly6C^hi^CCR2^+^CX_3_CR^−^_1_ monocytes (day 2–4) which can either transdifferentiate into or are replaced by reparative Ly6C^low/-^CCR2^−^CX_3_CR^+^_1_ monocytes (day 5–10). Both subsets can give rise to macrophages and/or dendritic cells (starting from d7 to 10) or modulate the activity of those cells already residing in the heart. Alternatively, those macrophages or dendritic cells which are essential for post ischemic remodeling and scar formation can also emerge from tissue resident precursors or bone-marrow-lineage independent macrophages. How natural killer cells crosstalk to those innate cells in the setting of MI is poorly understood.

Immediately after cardiac ischemia, different inflammatory signals recruit neutrophils to the infarct zone within 24 h, and monocytes/macrophages shortly thereafter (Figure 1). These homed leukocytes degrade extracellular matrix constituents and macromolecules released by injured cells. Neutrophils play an important role in the clearance of dead cardiac myocytes and their debris (Nahrendorf et al., 2007), release oxidants (mainly via their phagocyte type NADPH oxidase) and proteases and secrete mediators for inflammatory cell recruitment. Romson and coworkers could show in 1983, that depletion of these neutrophils in animals undergoing reperfused MI led to a marked decrease in infarct size, suggesting that a significant amount of myocardial injury induced by coronary artery occlusion followed by reperfusion may be neutrophil-dependent (Romson et al., 1983; Litt et al., 1989). The detrimental effects seem to be mediated by ICAM-1-dependent neutrophil-cardiomyocyte adhesion, a primary ligand of Mac-1 and CD11b/CD18 integrin. In an ICAM-1 ko mouse model it could be shown that there was less myocardial injury after MI (Metzler et al., 2001). Infarcted hearts modulate their chemokine expression profile over time, and sequentially and actively recruit Ly6C^hi^ and Ly6C^low^ monocytes. Ly6C^hi^ monocytes dominate early and exhibit phagocytic, proteolytic and inflammatory functions. In the later process Ly6C^low^ monocytes predominate. Consequently, Ly6C^hi^ monocytes digest damaged tissue, whereas Ly6C^low^ promote healing via myofibroblast accumulation, angiogenesis and deposition of collagen (Nahrendorf et al., 2007). In 2010 Panizzi et al. found that Ly-6Chi monocytosis disturbs resolution of inflammation in murine infarcts and consequently enhances left ventricular remodeling (Panizzi et al., 2010).

After recruitment in the infarcted territory, monocytes may differentiate into macrophages. Local upregulation of macrophage colony-stimulating factor (M-CSF) may play an important role in this process (Frangogiannis et al., 1998). Macrophage accumulation in the healing heart is regulated by the renin-angiotensin-aldosterone system. Angiotensin II (ATII) allows deployment of myelomonocytic cells via the ATII receptor type 1 (AT_1_R) from the spleen and directs them to the inflamed tissue (Swirski et al., 2009). ATII leads to release of the mineralocorticoid aldosterone from the adrenal glands. Aldosterone has pro-inflammatory effects via its mineralocorticoid receptor (MR) on cardiomyocytes. Selective MR blockade immediately after MI improved healing (Frantz et al., 2009), an effect that was blunted by macrophage depletion (Fraccarollo et al., 2008). Cardiomyocyte specific genetic ablation of the MR improved healing, partially via increased RANTES/CCR5 dependent chemotaxis of myelomonocytic cells supporting healing (Fraccarollo et al., 2011). This indicates, that recruitment of inflammatory cells not necessarily *impairs* healing, but is possibly also *required* to kick off the outbalanced sequence of cellular events involved in healing. Indeed, mice that were either chemically or genetically depleted of inflammatory myelomonocytic cells, died from cardiac rupture and atherothrombosis propably due to impaired wound healing (Frantz et al., 2013). The beneficial impact of macrophages on remodeling can partially be assigned to the release of soluble lipid mediators like resolvins, lipoxins and maresins. These factors have been shown to contribute to resolution of inflammation in general (Serhan, 2010). In ApoE^−/−^ mice fed a high fat diet, failure to form the inflammation-resolving lipids lipoxin A_4_, resolving D1 and protectin D1 accelerated atherosclerosis progression, while macrophage-specific overexpression of 12/15 lipoxygenase reduced atherosclerotic burden via the formation of these mediators (Merched et al., 2008). If resolution of MI related cardiac injury is improved by these mediators remains to be tested.

### Mutual activation of NK cells and monocytes in vascular dysfunction

Besides CD8^+^ T cells and T-bet^+^CD4^+^ T cells, Natural Killer (NK) cells are a major source of IFN-γ. In type 1 inflammatory immune responses including atherosclerosis and acute phase of MI, IFN-γ is known to play a major role in activating monocytes and macrophages (Boehm et al., 1997; Schroder et al., 2004) and driving them toward inflammatory phenotype (e.g., M1 macrophages or Ly6C^hi^ monocytes) (Abbas et al., 1996).

NK cells are key players in the innate immune response and produce a variety of cytokines such as IL-1beta, tumor necrosis factor alpha (TNFα), and IFN-γ and thereby participate in regulating innate and adaptive immunity (Yokoyama et al., 2004; Shereck et al., 2007). They represent a specialized lymphoid population, and act in an activating and inhibiting way by expressing different receptors (Moretta and Moretta, 2004). NK cells and monocytes/macrophages undergo reciprocal patterns of activation, that include receptor based cell-cell-contact triggered pathways mainly via CD154-CD40, 2B4, and NKG2D interactions and NKp46 conserved in a variety of species including mouse and human (for review, please see Michel et al., 2012). Of note, natural killer cell activation is in many aspects unique in humans as compared to mice, and even mice strains differ in the expression of certain surface markers and clusters of differentiation, like NK1.1, which is only found in C57BL/6 mice. In contrast, macrophage/NK cell interactions through soluble factors does not necessarily require cell-cell-contact for efficient signaling, which has gained growing attention in recent years. In a seminal work, Interleukin 12 (IL-12) was identified as *the* natural killer cell stimulatory factor (D'andrea et al., 1992) released by activated monocytes. Shortly thereafter, IL-12 and TNF-α released by activated macrophages were identified to be powerful inducers of IFN-γ by NK cells (Tripp et al., 1993).

It has been identified that macrophages can activate NK cells by producing interleukins such as IL-12 or IL-18 or through direct cell-to-cell contact (Aranha et al., 2005; Atochina and Harn, 2005). On the other side NK-cell-derived IFN-γ plays an important role in initializing the differentiation of monocytes into macrophages or dendritic cells, which are producers of IL-15, IL-12, and IL-18 (D'andrea et al., 1992; Welte et al., 2006; Goldszmid et al., 2012). By synergizing of IL-12 with IL-18, production of IFN-γ in NK cells increases, which is due to a positive feedback loop that represent an important mechanism in the early innate inflammatory response.

Mice deficient in the helix-loop-helix transcription factor inhibitor of differentiation (Id2(–/–)) which lack Langerhans cells, are defective in CD8^+^ T cells and dendritic cells as well as NK cells, were partially protected from ATII induced hypertensive damage. However, a clearcut effect of NK cells in this setting could not be delineated from these findings (Gratze et al., 2008). Interestingly, monocytes depend on T-bet for production of IL-12 and therefore for sufficient activation of NK cells to release IFN-γ (Soderquest et al., 2011). Kossmann et al. could recently show that angiotensin II-induced vascular dysfunction depends on vascular entry and IFN-γ production by NK cells. They identified IFN-γ was produced by NK cells in the aortic wall which initiate vascular oxidative stress, inflammatory cell recruitment and reciprocal innate immune cell activation in the vessel wall. In addition, T-bet deficient monocytes were not capable to induce ATII-dependent immune cell influx and NK-cell activation and depletion of NK-cells lead to protection from AT-II induced vascular dysfunction (Kossmann et al., 2013). This effect was equivalent to the effect of myelomonocytic cell depletion in LysM^iDTR^ mice in the same model of arterial hypertension that had been reported before (Wenzel et al., 2011), highlighting the importance of NK cell/monocyte interaction and mutual activation for induction and maintenance of vascular inflammation. More evidence supporting the importance of NK cells for vascular dysfunction was demonstrated by introducing the NK cell gene complex derived form C57BL/6 mice into the genome of BALB/C mice, rendering the strain as succeptible to vascular injury as the C57BL/6 mice in a hypertension model (Taherzadeh et al., 2010) and in a balloon injury model (De Vries et al., 2013).

Neither it is known, how these findings extrapolate to the situation in acute MI, nor if NK cells preferentially cooperate with Ly6C^hi^ (proinflammatory) or Ly6C^lo^ monocytes in vascular inflammation. Most pathways triggering an activating pattern of communication between NK cells and monocytes are hallmarked by TLR-ligands and proinflammatory cytokines like TNF-α, IFN-γ, IL-12, and IL-18 (Kloss et al., 2008; Vivier et al., 2008; Michel et al., 2012). This suggests, that NK cells preferentially cooperate with proinflammatory myelomonocytes in vascular inflammation and MI, or at least skews these cells toward this phenotype. This hypothesis, however, remains to be tested.

## Interaction of NK cells and monocytes in coronary artery disease and myocardial infarction

**Figure 2:**
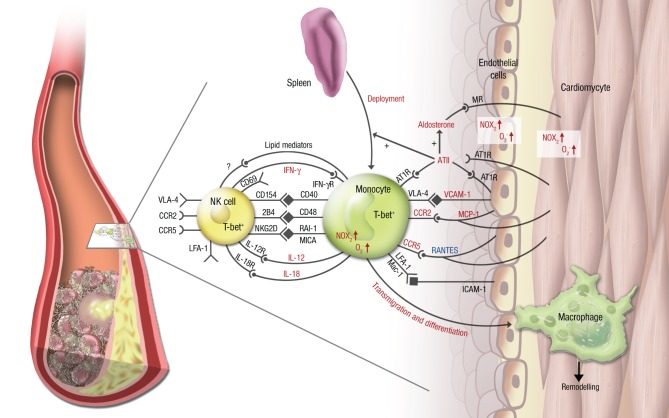
**Vascular inflammation, NK cell/monocyte crosstalk and angiotensin II in cardiac ischemic injury**. In cardiac ischemia reperfusion injury and remodeling after MI, vascular inflammation plays a central role. Monocytes transmigrate into the infarcted zone and transdifferentiate into macrophages, that can exert various functions in remodeling depending on the microenvironment provided by cytokines (e.g., IL-12, IL-18, IFN-γ), chemokines (e.g., RANTES/CCL5, MCP-1/CCR2), integrins (e.g., VCAM-1, ICAM-1) and soluble lipid factors like resolvins, lipoxins, and maresins. Signaling through angiotensin II and its downstream hormone aldosterone plays a central mechanistic role in this process. It leads to deployment of AT_1_R^+^ monocytes from the spleen to the infarcted area, increases (in red) or attenuates (in blue) chemokine and cytokine expression from the endothelium and from leukocytes, fosters monocyte transmigration and -differentiation and supports a reciprocal program of activation between NK cells and monocytes. While parts of this crosstalk have been partially investigated in this setting (e.g., the T-bet/IFN-γ/IL-12 pathway), most aspects of mutual NK cell/monocyte activation are not known in the setting of vascular inflammation and cardiac ischemic injury. This includes interaction between CD40 and CD154 (Bellora et al., 2010), CD48 and 2B4 (Nedvetzki et al., 2007) and NKG2D and retinoic acid inducible-1 (RAI-1) or members of the MHC class-I related chain (MIC) family of proteins, like MICA (Hamerman et al., 2004; Kloss et al., 2008). All of these are known to increase IFN-γ production by NK cells and CD69 expression on NK cells in infection or lipopoplysaccharide-driven models. Abbreviations: CD, cluster of differentiation; IL, interleukin; ATII, angiotensin II; AT1R, ATII receptor type 1; MR, mineralocorticoid receptor. Red letters: induced/activated by ATII/Aldosterone. Blue letters: attenuated by ATII/Aldosterone.

Little is known about the interaction of NK cells and monocytes/macrophages in the setting of atherosclerosis or acute MI. It was shown that NK cells are present in atherosclerotic plaques in humans as well as in mice (Whitman et al., 2004; Bobryshev and Lord, 2005), but the *in vivo* role of these immune cells in (coronary) atherosclerosis and MI is incompletely understood. It was shown *ex vivo* that NK cells produce more IFN-γ fostered by the CD48-2B4 axis (Figure 2) when co-cultured with monocyte derived dendritic cells, that had been exposed to oxidized LDL (Dong et al., 2011). This finding can at least in part explain the proatherogenic effects of NK cells.

Different research groups described a reduced NK cell activity in patients with CAD in comparison to healthy subjects (Jonasson et al., 2005; Hak et al., 2007). This effect was much more pronounced in patients in unstable conditions or acute coronary syndrome (Backteman et al., 2012; Hou et al., 2012).

NK cells show a biological variation over time and in a recently published 12-month follow-up study of patients with a coronary event, no evidence was found that reduction of NK cells was associated with aberrations in NK cells cell phenotype at any clinical stage of the disease. However, failure to reconstitute NK cell levels was associated with a persistent low-grade inflammation, suggesting a protective role of NK cells in CAD (Backteman et al., 2012). The mechanism why there is a deficit of NK cells in CAD is not identified yet, but an increased apoptosis of these cells in CAD patients has been reported (Li et al., 2008). On the other side data from experimental studies demonstrated that NK cells infiltrate the vessel wall and promote atherosclerotic lesion development (Whitman et al., 2004). A recently published work of Selathurai and coworkers demonstrated evidence that NK cells are atherogenic and their production of perforin and granzyme B contributes to atherosclerosis and the expansion of necrotic cores (Selathurai et al., 2014) representing vulnerable lesions prone for atherothrombosis as in MI.

RAAS signaling might play an important role in promoting mutual activation of NK cells and myelomonocytic cells, given its key role in hypertension, atherogenesis and MI (summarized in Figure 2). Expression and activity of many chemokines, integrins and cytokines and its receptors (e.g., MCP-1/CCR2, IFN-γ/IFN-γR, VCAM-1/VLA-4) as well as pro-atherogenic enzymes (e.g., NADPH oxidase) were shown to be driven by ATII/aldosterone on monocytes and the vessel, but not yet on NK cells, although their battery of signaling structures (e.g., CCR2, CCR5, LFA-1, VLA-4) share many similarities with monocytes (Figure 2).

## Conclusion

Natural killer cells and monocytes/macrophages with their mutual programs of interactions are an important asset to the innate immune response. Its role in vascular inflammation, atherosclerosis, coronary artery disease and MI is now starting to catch interest from immunologist and vascular biologist (for scheme, see Figure 2). The molecular and physiologic interactions are nevertheless poorly explored and incompletely understood, although some potential targets have arisen from experimental research, that might be attractive for therapeutic targeting.

## Sources of funding

Maike Knorr and Philip Wenzel are supported by the German Federal Ministry of Education and Research (BMBF 01EO1003). Philip Wenzel is supported by grants from the German Research Foundation (DFG 4361/3-1 and 4361/4-1) related to this work.

### Conflict of interest statement

The authors declare that the research was conducted in the absence of any commercial or financial relationships that could be construed as a potential conflict of interest.
